# Divergent responses of human intestinal organoid monolayers using commercial *in vitro* cytotoxicity assays

**DOI:** 10.1371/journal.pone.0304526

**Published:** 2024-06-10

**Authors:** Miranda A. Lewis, Ketki Patil, Khalil Ettayebi, Mary K. Estes, Robert L. Atmar, Sasirekha Ramani

**Affiliations:** 1 Department of Molecular Virology & Microbiology, Baylor College of Medicine, Houston, Texas, United States of America; 2 Department of Medicine, Baylor College of Medicine, Houston, Texas, United States of America; Università degli Studi della Campania, ITALY

## Abstract

*In vitro* models, such as primary cells and continuous cell lines routinely used for evaluating drug candidates, have limitations in their translational relevance to human diseases. Organotypic cultures are increasingly being used to assess therapeutics for various cancers and infectious diseases. Monitoring drug cytotoxicity in cell cultures is crucial in drug development, and several commercially available kits for cytotoxicity assessment offer distinct advantages and limitations. Given the complexity of organoid cultures, including donor-driven variability, we investigated drug-treated, tissue stem cell-derived human intestinal organoid responses with commonly used cell cytotoxicity assay kits. Using seven different compounds, we compared the cytotoxicity assay performance of two different leaky membrane-based and two metabolism-based assays. Significant variability was seen in reported viability outcomes across assays and organoid lines. High baseline activity of lactate dehydrogenase (LDH) in four human intestinal organoid lines required modification of the standard LDH assay protocol. Additionally, the LDH assay reported unique resilience to damage in a genetically-modified line contrasting results compared to other assays. This study highlights factors that can impact the measurement of cell cytotoxicity in intestinal organoid models, which are emerging as valuable new tools for research and pre-clinical drug testing and suggest the need for using multiple assay types to ensure reliable cytotoxicity assessment.

## Introduction

Determining cell viability is a cornerstone in various biological disciplines. In the realm of drug discovery, *in vitro* models are indispensable tools, especially in the early stages of preclinical evaluation. Particularly for antiviral and pharmacological research, *in vitro* models provide essential data on drug interactions, metabolism, and potential toxicity. The traditional *in vitro* models, such as primary cells and continuous cell lines derived from human or animal tissues, have significantly advanced our understanding of drug interactions at a cellular level. Yet, the journey from bench to bedside is plagued with challenges, as evidenced by a success rate of less than 10% for drugs moving from Phase I trials to market approval [1]. Many compounds showing promise in conventional *in vitro* settings fail to translate into successful clinical outcomes due to issues like lack of efficacy or unforeseen toxicity in humans [2, 3]. These statistics underscore the critical need for more accurate and reliable preclinical models to predict human responses.

The majority of standard laboratory cell lines are cancer-derived or are primary cells that have been transformed to grow indefinitely. Continuous cell lines such as Caco-2, despite their widespread use, present limitations in accurately representing human intestinal metabolism due to their origin from cancer cells and altered expression profiles of drug metabolizing enzymes and transporters [4, 5]. Recent advancements in preclinical models have brought the use of human organoid cultures to the forefront [6]. Human intestinal organoids (HIOs), reflecting the diverse cell types and complex architecture of the intestinal epithelium, are an attractive new model to enhance the predictive accuracy of drug testing for gastrointestinal infections and diseases. These innovative models, derived from human intestinal stem cells, are more representative of the human intestine compared to traditional continuous cell lines such as Caco-2 cells. This is crucial considering the pivotal role of the gastrointestinal (GI) tract in the absorption, distribution, metabolism, and excretion (ADME) of orally administered compounds [7–9].

The emergence of HIOs represents a significant step forward in mimicking human intestinal physiology, offering a potentially more reliable platform for evaluating drug absorption and toxicity [6]. However, the application of HIOs in drug testing is still in its nascent stages. There are inherent benefits and challenges to this model such as the heterogeneity in cell composition, varied plating formats, derivation from different intestinal segments, and the use of diverse patient donors that can lead to response variability across studies [10, 11]. Such variability raises the question of whether different commercially available cytotoxicity assays might also yield divergent results when applied to HIOs. Recognizing this knowledge gap, our study aimed to evaluate drug cytotoxicity in different HIO lines, including genetically-modified ones, focusing on jejunal HIOs cultured as monolayers in 96-well plates. This approach is particularly suited for high-throughput studies and allows for direct luminal exposure to compounds. By comparing various cytotoxicity assays, we sought to better understand HIO performance in drug toxicity studies and determine if the assay choice results in variability. This study contributes to the evolution of preclinical models and has the potential to reduce the reliance on animal testing, thereby advancing the field of drug development.

## Materials and methods

### HIO cultivation and seeding

Three-dimensional (3D) cultures of jejunal HIO cultures were obtained from the organoid core at Baylor College of Medicine (BCM). The J2 and J4 HIO lines originally were developed from biopsy specimens collected during bariatric surgery, as described previously [12, 13]. Genetically-modified lines include a signal transducer and activator of transcription 1 knock-out of J2 (J2 *STAT1*^*-/-*^), a fucosyltransferase 2 knock-out of J2 (J2 *FUT2*^*-/-*^) and fucosyltransferase 2 knock-in of J4 (J4 *FUT2*^*-/-/FUT2*^) and were generated as described previously [14, 15]. 3D cultures of HIOs suspended in Matrigel were maintained in L-WRN proliferation medium (prepared from cell line ATCC CRL-3276 grown in Dulbecco modified Eagle medium (DMEM-F-12 (Invitrogen, 12634–028)) supplemented with 20% HyClone™ Fetal Bovine Serum (FBS; Cytiva, SH30071.03) until processing into monolayer cultures. Monolayer cultures were seeded into 96-well plates using a 1:1 ratio of IntestiCult™ Organoid Growth Medium (OGM) Human Basal Medium (StemCell Technologies, 100–0190) and Organoid Supplement (StemCell Technologies, 100–0191) supplemented with 10 μM ROCK inhibitor Y-27632 (Sigma, Y0503) for 24 hours. For experiments evaluating cytotoxicity assays in 3D HIOs, the cells were plated as 3D cultures in 96-well plates and were suspended in 5 μL of Matrigel. Differentiation medium composed of a 1:1 ratio of Intesticult™ OGM medium and complete medium without growth factors (CMGF-, advanced DMEM-F-12 prepared with 1X GlutaMAX (Gibco, 35050–061) and 10 mM HEPES (Gibco, 15630–080)) [16] was then added to the HIOs for 3 and 5 days for 3Ds and monolayers, respectively. Cells were counted at seeding, when indicated, using trypan blue (Gibco, 15250061) and a TC20™ Automated Cell Counter (BioRad, 1450102).

### Drug treatment of HIOs

United States Pharmacopeia (USP)-grade ritonavir (Sigma, 1604803-200MG), valacyclovir (Sigma, 1707839-200MG), and ribavirin (Sigma, 1602706-200MG) were serially diluted in 0.5 log_10_ increments in vehicle (≥99.7% pure DMSO (Sigma, D2650) for ritonavir or milliQ H_2_O for valacyclovir and ribavirin). Additional compounds include nitazoxanide (Sigma, 1463960), auranofin (Sigma, A6733-10MG), oligomycin A (Sigma, 75351-5MG), each dissolved in DMSO, and digitonin (Santa Cruz Biotech, sc-280675A) dissolved in ethanol at a single dose. Final dilutions of the compounds in vehicle were added to differentiation medium. Compounds in media were then added onto wells of a given HIO line in triplicate for monolayers or 8–10 replicates for 3Ds and incubated at 37°C for 24 h.

### Assessment of HIO viability

Cell viability was determined using different commercial kits: CytoTox 96® Non-Radioactive Cytotoxicity Assay (LDH; Promega, G1780), CellTiter 96® Aqueous One Solution Cell Proliferation Assay ([3-(4,5-dimethylthiazol-2-yl)-5-(3-carboxymethoxyphenyl)-2-(4-sulfophenyl)-2H-tetrazolium] or MTS; Promega, G3580), CellTox™ Green Cytotoxicity Assay (CellTox; Promega, G8742), and CellTiter-Glo® 2.0 Cell Viability Assay (CellTiter-Glo; Promega, G9242). HIO cells were seeded onto clear 96-well plates (Costar, 3595) for the LDH and MTS assays and black 96-well plates (Greiner Bio-One, 655086) for the CellTox and CellTiter-Glo assays, respectively. Cells were treated with vehicle and compound in differentiation media. After 24 hours of treatment of a compound, cell viability was determined. The LDH assay was performed either according to the manufacturer’s protocol or with a modification wherein supernatants were diluted in BCMd media (prepared as described in Ettayebi et al. [16]), where stated. Experiments using the MTS assay were performed using the manufacturer’s protocol and multiplexed with the modified LDH assay method. The CellTox assay was multiplexed with the CellTiter-Glo assay according to the manufacturer’s protocol. Microplate-based cell viability assays were read using a SpectraMAX M5 spectrophotometer. For flow cytometry, monolayers were trypsinized with trypLE (Gibco, 12605028) for 10–30 min at 37°C until single cells were observed. CMGF- supplemented with 10% FBS was added after trypsinization. Three wells of each condition were pooled and centrifuged at 300g for 5 min. Cells were stained first with the LIVE/DEAD™ Fixable Aqua Dead Cell Stain Kit (Invitrogen, L34965) according to the manufacturer’s protocol. The cells were then stained with 10 μg/mL propidium iodide (Invitrogen, P1304MP) and run on an Attune NxT flow cytometer. Gating strategy and analysis are shown in the supplementary data [(S1 Fig in S1 File)]. For epifluorescence images, monolayers treated for 24 h were stained with 10 μg/mL propidium iodide for 10 minutes followed by 3 washes with CMGF-. 4% Paraformaldehyde (Electron Microscopy Sciences) was then added for 15 minutes followed by 3 washes with PBS. Monolayers were then permeabilized using 0.5% Triton-X 100 for 15 minutes, then washed with PBS and stained using DAPI (Invitrogen R37606). After incubating, monolayers were washed 3 times with PBS. Cells were imaged at 20X magnification using an Olympus microscope.

### Statistical analyses

Three technical replicate wells were used for each treatment performed on HIOs as monolayers. For 3D HIOs, 8–10 replicate wells were used. Data averaged from each condition were then pooled across repeated experiments as stated in the figure legends. Untreated cells in media served as the background to subtract from all samples. Percent cytotoxicity and viability was calculated as a ratio of samples to the positive control. Values outside of the upper range of 100% cytotoxicity and lower range of 0% for viability were set to 100 and 0, respectively. Percent cytotoxicity values were then converted as 100 minus percent cytotoxicity to compare with percent viability results. For comparing cytotoxicity assay responses from drug treatment on HIOs, two-way ANOVA and Tukey post-hoc multiple comparisons tests were performed. Non-linear regression analysis was performed on LDH enzyme activity and viability data to determine the 50% effective and cytotoxic concentration (EC_50_ and CC_50_, respectively). For EC_50_ calculations, a 4-parameter logistic non-linear regression was used with the top and bottom OD_490_ values constrained to <3.5 and >0, respectively. For CC_50_ calculations, the [Agonist] vs. response–Find ECanything non-linear regression formula was used in GraphPad Prism, with the bottom value constrained to 0. Statistical analyses were performed using GraphPad Prism 9.5.0.

## Results

### High lactate dehydrogenase activity of HIOs necessitates modification of LDH assay

**Fig 1:**
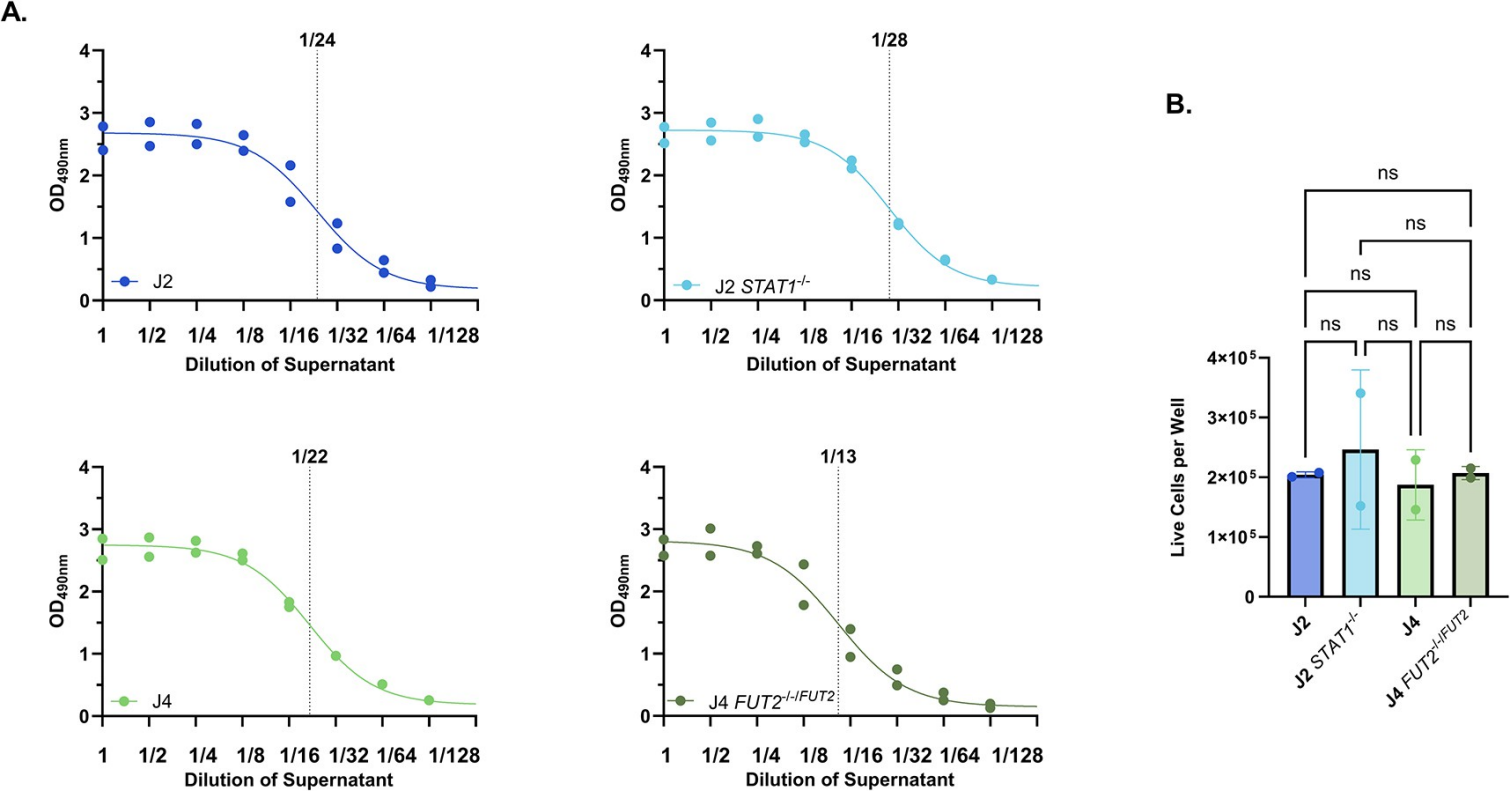
HIOs exhibit high basal LDH activity. A) J2, J2 *STAT1*^*-/-*^, J4 and J4 *FUT2*^*-/-/FUT2*^ HIOs were lysed with 0.8% Triton-X 100. Supernatants were diluted 2-fold and used in an LDH reaction using the CytoTox 96® Kit. Non-linear regression. B) HIO monolayers were trypsinized and live cells were counted using trypan blue. One Way ANOVA and post hoc Tukey test. ns = not significant. Data are compiled from n = 2 experiments.

Due to its cost-effectiveness, simplicity, and wide-spread use, we first evaluated the lactate dehydrogenase assay (LDH) for cytotoxicity assessment. Untreated J2, J2 *STAT1*^*-/-*^, J4, and J4 *FUT2*^*-/-/FUT2*^ HIO monolayers were lysed using the LDH assay kit lysis buffer to release total LDH enzyme followed by a 30-minute reaction using the kit’s substrate. Following this standard protocol, we observed that the total LDH activity from undiluted supernatants yielded OD_490_ readings upwards of 2.5. Two-fold dilutions of the supernatants in BCMd media prior to the assay reaction led to a linear range of OD_490_ values between 0.5 and 2.5 (Fig 1A). Analysis using non-linear regression showed the 50% effective concentration (EC_50_) for LDH enzyme activity to range between 1/22 and 1/28 dilution for J2, J2 *STAT1*^*-/-*^, and J4 monolayers (Fig 1A). The J4 *FUT2*^*-/-/FUT2*^ HIO line required less dilution (1/13) to reach 50% activity (Fig 1A), suggesting either lower LDH enzyme quantities or reduced LDH enzymatic activity in this HIO line. Cell count differences were not responsible for differences of baseline LDH activity of different cultures as the live cell counts were similar at seeding for the four HIO lines (Fig 1B). These results demonstrated that dilution of HIO supernatants is necessary to get within the linear range for measuring maximal enzyme activity for all lines tested and highlights potential variances in LDH enzyme activity in different cell lines.

**Fig 2:**
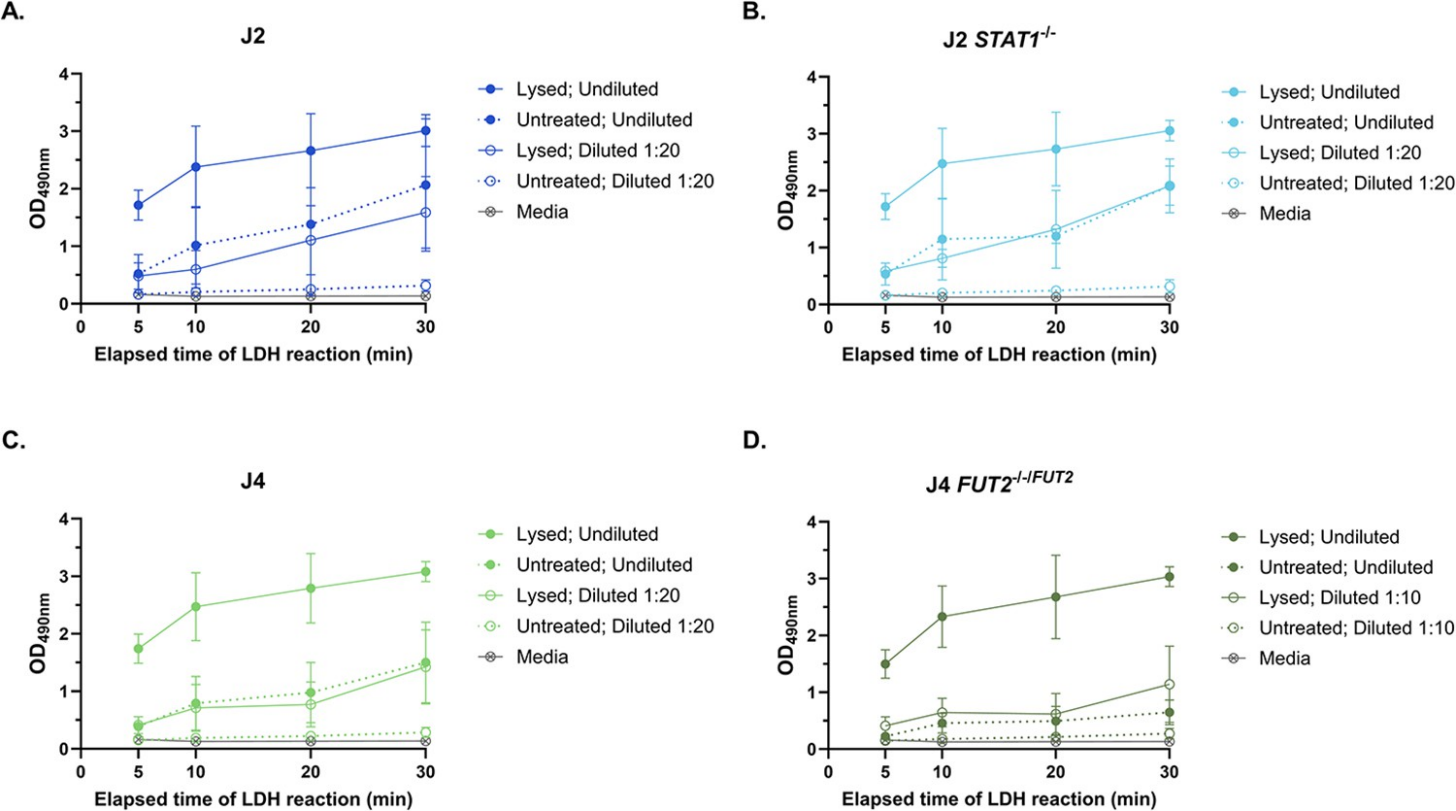
Modification of the LDH assay yields OD values in the linear range of the assay. HIOs were lysed with 0.8% Triton-X 100 to release total LDH into the supernatant. A) J2, B) J2 *STAT1*^*-/-*^, and C) J4 samples were used undiluted and diluted 1:20, while D) J4 *FUT2*^*-/-/FUT2*^ samples were used undiluted and diluted 1:10. Reactions were stopped at 5, 10, 20, and 30 minutes and the absorbance at 490 nm was recorded. Data are compiled from n = 3 experiments.

After determining that HIOs exhibit high basal LDH activity, we investigated two strategies to adapt the LDH cytotoxicity assay to achieve results within the linear range of the assay: 1) shortening the LDH reaction time and 2) using the recommended reaction time but comparing the OD_490_ of undiluted to diluted HIO supernatants. We used dilutions of 1:20 for J2, J2 *STAT1*^*-/-*^ and J4 and 1:10 for J4 *FUT2*^*-/-/FUT2*^ based on their EC_50_ determined previously (Fig 1), rounding to a multiple of 10 for simplicity of calculations. The LDH activity (as measured by OD_490_) of undiluted supernatants of lysed HIO monolayers yielded readings in the upper plateau of >2.5 OD_490_ by 10 minutes of reaction time for all four HIO lines, suggesting that shortening the LDH reaction time to less than 10 minutes will be required to achieve values within the linear range (Fig 2). In contrast, diluted supernatants consistently yielded OD_490_ values within the appropriate range of >0.5 and <2.5 OD_490_ at 10 minutes and beyond. Further, untreated HIO monolayers demonstrate OD_490_ readings above 0.5 in undiluted supernatants across all time points, whereas diluted, untreated monolayers remained below the assay’s linear range. These data indicate that diluting HIO supernatants is essential to obtain OD readings within the linear range of the assay, thereby mitigating potential data skewing due to the high LDH baseline enzymatic activity in HIO monolayers.

### Variability in cytotoxicity is assay-, compound-, and line-dependent

Next, we evaluated the performance of four different commercial cytotoxicity assays with different mechanisms of assay readout: CellTox, MTS, CellTiter-Glo, and LDH. Both the LDH and CellTox assays rely on damaged or “leaky” membranes in which the active reagent is used as a proxy to measure cytotoxicity. We used a modified LDH assay by testing diluted supernatants as described above (Fig 2). The CellTiter-Glo and MTS, a modified version of the (3-(4,5-dimethylthiazol-2-yl)-2,5-diphenyltetrazolium bromide (MTT) assay, measure viability through healthy cells that have adequate ATP content or are capable of metabolizing the kit reagent. Considering that a prior study reported significant discrepancies in cytotoxic concentration 50 (CC_50_) values across four distinct assays for chloroquine and sodium azide [17], we investigated the performance of these assays in our study for antiviral compounds. As another measure of cytotoxicity, we assessed cell death using flow cytometry with propidium iodide and a live/dead dye dependent on permeabilized membranes of dead cells. To quantitatively compare the assays, we estimated the CC_50_ of drug treatment for each HIO line.

**Fig 3:**
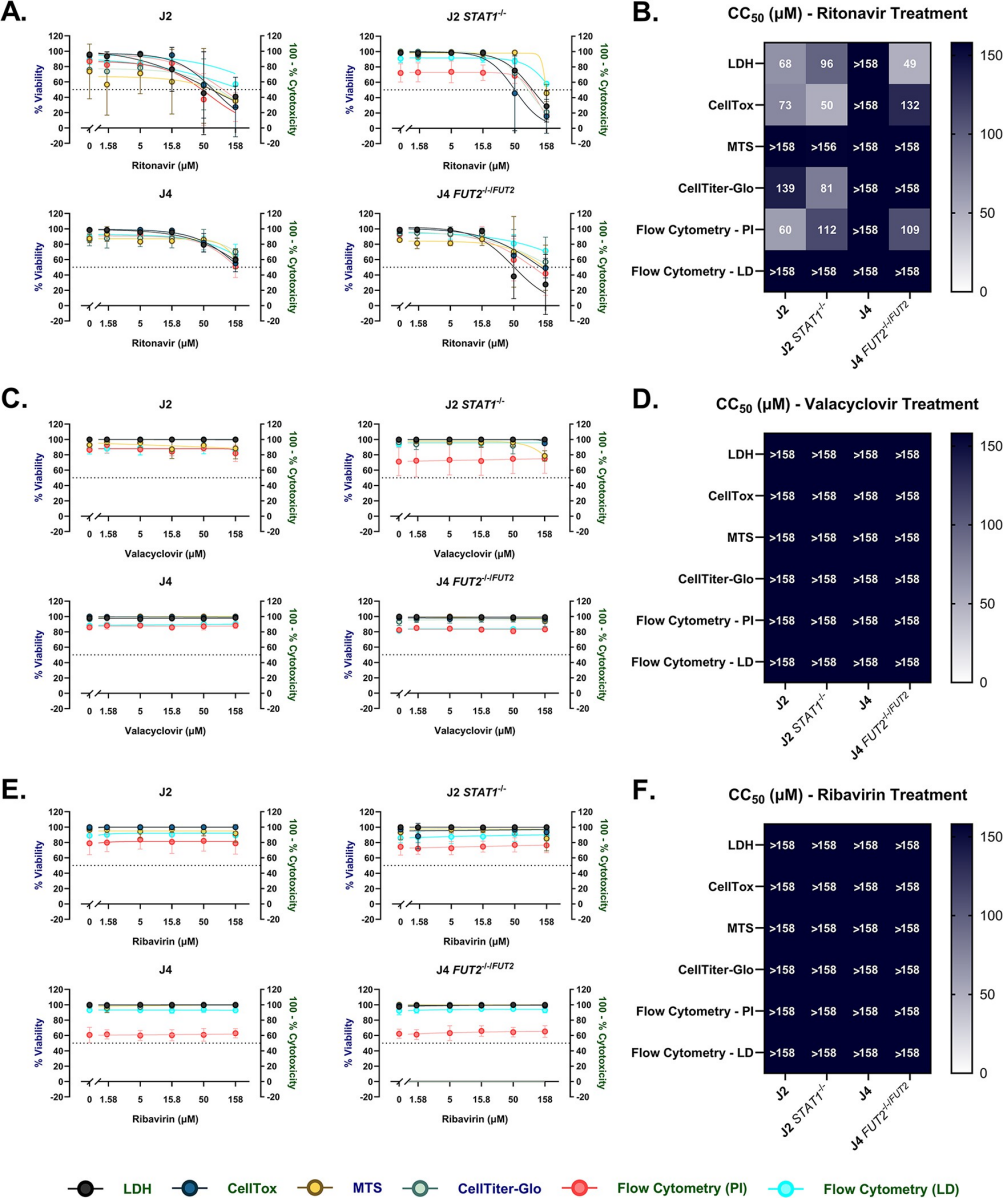
A cytotoxic antiviral compound demonstrates high variability in estimated CC_50_ among different HIO lines. J2, J2 *STAT1*^*-/-*^, J4 and J4 *FUT2*^*-/-/FUT2*^ HIOs were treated with a range of doses with the following antivirals: A) a protease inhibitor (ritonavir), C) DNA polymerase inhibitor (valacyclovir), and E) RNA-dependent RNA polymerase inhibitor (ribavirin). Assays were done 24 h post treatment. 100 minus percent cytotoxicity was measured by the CytoTox 96® Non-Radioactive Cytotoxicity Assay and CellTox™ Green Cytotoxicity Assay. Percent viability was measured by the CellTiter 96® AQueous One Solution Cell Proliferation Assay and the CellTiter-Glo® 2.0 Cell Viability Assay. Viability was validated by quantitation using flow cytometry using propidium iodide and LIVE/DEAD™ dyes. CC_50_s estimated by non-linear regression for B) ritonavir, D) valacyclovir, and F) ribavirin are plotted. The data from LDH, CellTox, and flow cytometry assays are plotted on the right y-axis (green) and data from the MTS and CellTiter-Glo assays are plotted on the left y-axis (blue). Data are compiled from n = 2 experiments.

The cytotoxicity of three antivirals with different mechanisms of action was determined. Ritonavir, a viral protease inhibitor, showed a dose-dependent decrease in viability (increased cytotoxicity) among all four HIO lines tested (Fig 3A). Upon treatment with ritonavir, the estimated CC_50_ from the LDH assay was similar to that from the CellTox assay for J2 at 68 and 73 μM, respectively (Fig 3B). In contrast, the metabolism-based assays, MTS and Cell-Titer Glo, resulted in higher CC_50_ of values of >158 and 139 μM, respectively. Given that the highest concentration of ritonavir tested did not decrease viability below 50% for J2 by the MTS assay, an accurate CC_50_ could not be estimated and was designated >158 μM. In J2 *STAT1*^*-/-*^, there was a near 2-fold difference in CC_50_ between plate-based assays of the same mechanism (LDH compared to CellTox and MTS compared to CellTiter-Glo). In comparison to the other HIO lines, ritonavir caused less cytotoxicity or a lower loss of viability for the J4 HIO line by all methods of cytotoxicity quantitation. The CC_50_ values were calculated as >158 μM for all four assays. In J4 *FUT2*^*-/-/FUT2*^, ritonavir exhibited less cytotoxicity as demonstrated by CC_50_s 132 μM and above with the CellTox, MTS, and CellTiter-Glo assay. By contrast, the CC_50_ based on the LDH assay for J4 *FUT2*^*-/-/FUT2*^ ritonavir treated HIOs was more than 2-fold lower at 49 μM, signifying a potential difference in reporting viability in this assay. In evaluating the performance of these assays in 3D HIOs, cytotoxicity due to ritonavir also demonstrated variable outcomes, with large differences between the LDH assay and other assays in J4 *FUT2*^*-/-/FUT2*^ (S2 Fig in S1 File). When using flow cytometry on HIO monolayers, propidium iodide staining measured ritonavir-associated cytotoxicity results similarly to the LDH and CellTox assays. However, the LIVE/DEAD stain did not report viability below 50% for any of the lines tested. As a result, the CC_50_ could not be estimated with this assay. This result was similar to the MTS results for this compound. Using fluorescent microscopy, we did not see a high number of propidium iodide-positive cells (S3 Fig in S1 File). However, the highest concentration of ritonavir caused the cells to slough off the plates after the washes, as depicted by the lack of DAPI staining.

Neither valacyclovir, a viral DNA polymerase inhibitor, nor ribavirin, a viral RNA-dependent RNA polymerase inhibitor, were cytotoxic at the concentrations evaluated (Fig 3C and 3E). Therefore, the CC_50_ was designated >158 μM for all HIO lines (Fig 3D and 3F). Although lower viability with these two compounds was reported by the two measures of flow cytometry, the vehicle control showed similar percent viability to the increasing doses of valacyclovir and ribavirin tested, indicating no detected cytotoxicity due to the compound. Collectively, the four microplate-based assays exhibited significant variation in response to a cytotoxic antiviral ritonavir, while flow cytometry failed to validate a singular plate-based assay for viability measurement.

**Fig 4:**
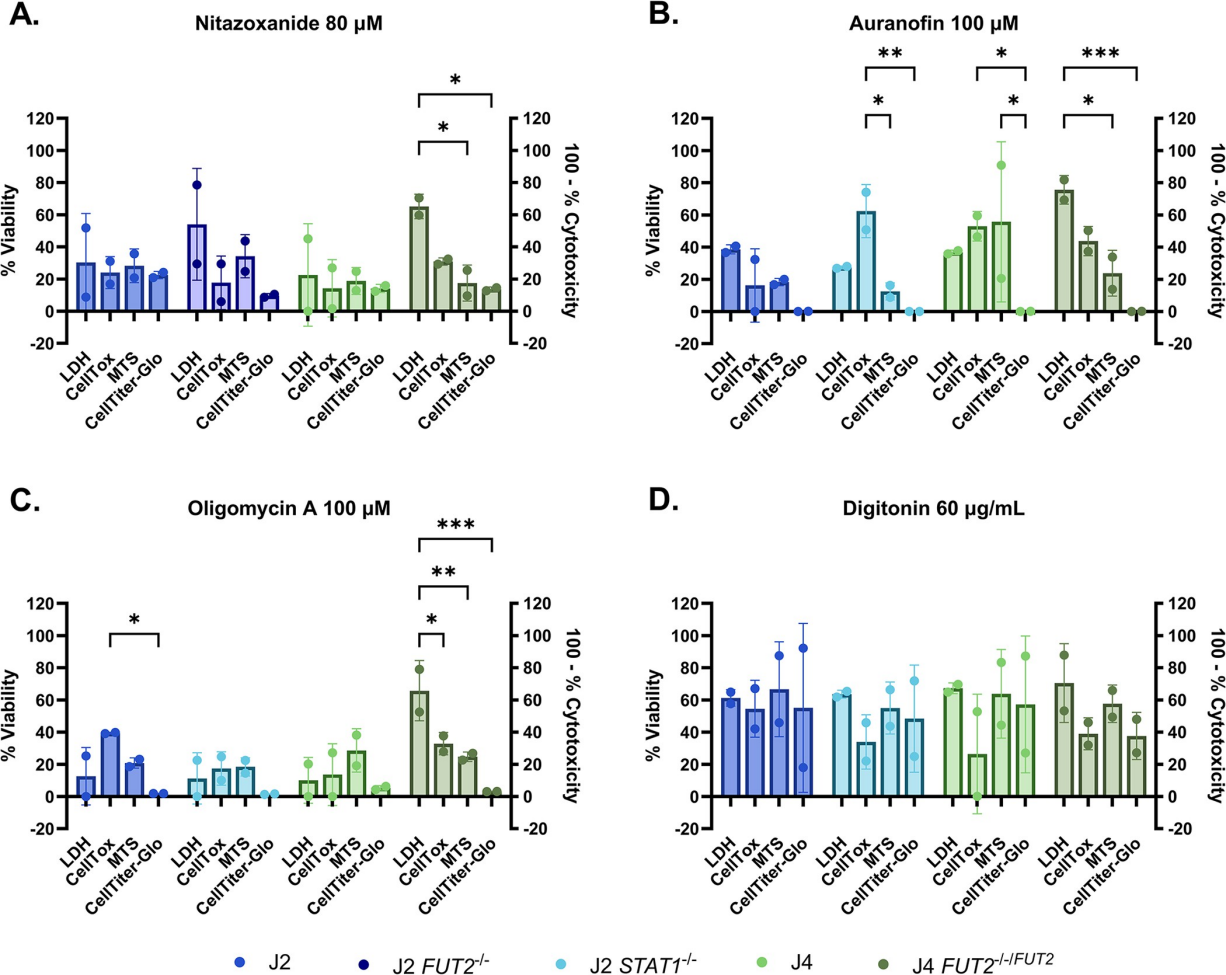
Assay-specific responses in HIOs demonstrate compound-dependent cytotoxicity patterns. J2, J2 *STAT1*^*-/-*^ or J2 *FUT2*^*-/-*^, J4 and J4 *FUT2*^*-/-/FUT2*^ HIOs were treated with the following compounds: A) 80 μM nitazoxanide, B) 100 μM auranofin, C) 100 μM oligomycin A, and D) 60 μg/mL digitonin. Assays were done 24 h post treatment. 100 minus percent cytotoxicity was measured by the CytoTox 96® Non-Radioactive Cytotoxicity Assay and CellTox™ Green Cytotoxicity Assay. Percent viability was measured by the CellTiter 96® AQueous One Solution Cell Proliferation Assay and the CellTiter-Glo® 2.0 Cell Viability Assay. Two Way ANOVA and post hoc Tukey test. Data are compiled from n = 2 experiments.

We further measured the cytotoxicity of four additional compounds with each of the four commercial cytotoxicity assays. Single concentrations of nitazoxanide (anti-parasitic), auranofin (anti-rheumatic), oligomycin A (ATP synthase inhibitor) and digitonin (a detergent) were evaluated. Each compound was tested at a concentration that resulted in a 20% or greater decrease in viability in all assays for each HIO line evaluated (Fig 4). Upon treatment of J2, J2 *FUT2*^*-/-*^, and J4 HIOs with 80 μM nitazoxanide, the reported viability was below 50% and outcomes from the different cytotoxicity assays were not significantly different from each other. However, in J4 *FUT2*^*-/-/FUT2*^ HIOs, 80 μM nitazoxanide treatment resulted in 70% viability with the LDH assay, displaying significant differences from the metabolism-based assays (Fig 4A). Treatment of J2 *STAT1*^*-/-*^, J4, and J4 *FUT2*^*-/-/FUT2*^ HIOs with 100 μM auranofin resulted in highly varied viability responses (Fig 4B). The CellTiter-Glo assay yielded 0% viability among the four lines tested, contrasting the results from other assays. Among J2 *STAT1*^*-/-*^ HIOs, auranofin treatment yielded higher reported viability in the CellTox assay versus the significantly lower results from MTS and CellTiter-Glo metabolism-based assays. In J4 *FUT2*^*-/-/FUT2*^, the LDH assay results were significantly different from the metabolism-based assays. Using oligomycin A, there was less than 40% viability in J2, J2 *STAT1*^*-/-*^, and J4 HIOs (Fig 4C). Among the different cytotoxicity assays in J2 *STAT1*^*-/-*^ HIOs, the CellTox assay reported significantly higher percent viability than the reported viability from the CellTiter-Glo assay. In J4 *FUT2*^*-/-/FUT2*^, the LDH assay yielded viability at 70% which is significantly higher than the other three assays in response to oligomycin A treatment. Unexpectedly, there were no significant differences in the response to 60 μg/mL digitonin treatment across the four assays in J2, J2 *STAT1*^*-/-*^, J4, and J4 *FUT2*^*-/-/FUT2*^ HIOs. Overall, these additional compounds exhibited substantial variability in reported viability across assays, with the LDH assay showing significant variation in reporting viability specifically in the genetically-modified J4 *FUT2*^*-/-/FUT2*^ HIO line for three out of four compounds.

## Discussion

Assessment of cytotoxicity is a cornerstone of drug development, distinguishing between a drug’s intended therapeutic action and undesirable cytotoxic effects on host cells. HIOs represent an advanced platform for preclinical drug evaluation and therapeutic research based on their proven physiological relevance in modeling human phenotypes and range of responses owing to their ability to be derived from various segments and from different individuals, coupled with their alignment with precision medicine and suitability for high-throughput drug screening. Several different cytotoxicity assays have been reported using HIOs and our research demonstrates that the choice of cytotoxicity assay impacts the interpreted viability of these cultures post-drug exposure.

Each of the commercially available assays works on a different principle and we observed several advantages and limitations to each assay that are summarized in Table 1. For example, the LDH and MTS assays offer a cost advantage per reaction and are colorimetric assays that utilize widely available spectrophotometers and are thus highly accessible. In contrast, the CellTox and CellTiter-Glo assays offer multiplexing capabilities and wider assay ranges, thus enhancing sensitivity, but necessitate specialized plate-readers. We utilized these four assays in tandem to measure the cytotoxicity with several drugs and observed differences in results across assays. An important observation we made during our studies is that moderate cytotoxicity in HIOs can cause intact monolayers to detach from the well, which potentially impacts percent viability outcomes in assays relying on intact cells such as the CellTox assay. Furthermore, variations in protocol and incubation times or intended outcomes influence preferences; for instance, the CellTox and CellTiter-Glo assays may be preferred because they require fewer steps and shorter reaction times. Although flow cytometry is highly quantitative, it is very labor intensive for higher throughput screening and necessitates specialized training and equipment.

**Table 1:** Summary of benefits and limitations of *in vitro* cytotoxicity and viability assays used in this study.

Assay	Assay Mechanism	Limitations	Benefits
LDH	Leaky Membrane; LDH Activity	Low sensitivity, multi-step protocol, protocol requires modification in HIOs	Low cost, capacity to test same sample over time, does not expend samples, colorimetric spectrophotometer is widely available
CellTox	Leaky Membrane; DNA staining	Potential inaccuracies with migrating cells, expends samples, higher cost	High sensitivity, quick protocol
MTS	Metabolism; mitochondrial activity	Low sensitivity, expends samples	Low cost, colorimetric spectrophotometer is widely available
CellTiter-Glo	Metabolism; ATP content	Expends samples, higher cost	High sensitivity, quickest protocol, homogenizes solution
Flow Cytometry	Leaky Membrane; DNA staining & Amine binding	Requires specialized training and equipment	Highly sensitive and quantitative

The LDH assay is among the most widely used methods to measure cytotoxicity. In our study, we observed that the HIOs have high levels of basal LDH activity, necessitating diluting supernatants to obtain OD values in the linear range of the assay. We verified this by comparing the OD readings of diluted supernatants to undiluted supernatants that were subject to a lower reaction time. We observed that the ODs of diluted supernatants after 30 minutes of reaction were comparable to ODs of undiluted supernatants that had reactions stopped at 5 minutes. While both conditions result in OD values within the assay’s linear range, we recommend the dilution method, particularly when processing a large number of samples. Given differences between HIO lines as seen with lower LDH activity of J4 *FUT2*^*-/-/FUT2*^ cells, it is also important to dilute supernatants relative to the enzymatic capability of a specific HIO line. These modifications are essential to establish a reliable method to standardize assaying cytotoxicity in HIOs using the LDH assay.

When examining the cytotoxicity that ritonavir causes in the HIOs, the CellTox assay results were in agreement with the LDH assay responses for the J2 and J2 *STAT1*^*-/-*^ lines whereas the metabolism-based assays reported about twice the concentration for the estimated CC_50_. Ritonavir, originally an HIV protease inhibitor is now often used clinically to boost plasma concentrations of the co-administered drugs given its ability to irreversibly inhibit the drug metabolizing enzymes, CYP3A4 and CYP3A5 [18] and the drug efflux pump, P-glycoprotein [19]. A potential reason for the observed variations in cytotoxicity in J2 and J2 *STAT1*^*-/-*^ caused by ritonavir might be linked to the role of CYP enzymes in vital cellular processes, including the metabolism of cholesterol and fatty acids, which are crucial for maintaining cellular homeostasis and membrane integrity [20]. Although fatty acids are used for cellular metabolism, there are likely enough supplements present in the culture media for the HIOs to rely on other macromolecules for ATP production even in the presence of ritonavir’s effects on CYP enzymes.

For ritonavir treatment of J4 *FUT2*^*-/-/FUT2*^, only the LDH assay differed from the CC_50_s of the other microplate assays. When examining additional compounds, we saw more pronounced differences in viability when comparing the LDH assay to the metabolism-based assays for this genetically-modified J4 *FUT2*^*-/-/FUT2*^ line. Upon examining a single concentration of nitazoxanide, auranofin, and oligomycin A, the LDH assay consistently showed substantially higher viability for the J4 *FUT2*^*-/-/FUT2*^ line in comparison to the metabolism-based assays; these differences are not observed in the parental J4 HIO line. It is not evident whether these differences are attributed to inherently low LDH enzyme activity, better preserved membrane integrity in these cells, or if the J4 *FUT2*^*-/-/FUT2*^ cells are generally less susceptible to cytotoxic effects. It is also possible that genetic modification of HIOs influences these factors; however, these differences are not observed in the genetically-modified STAT1 knock-out line of J2. These results indicate a need for deeper understanding of the physiology of these genetically modified lines and of understanding differences between donors.

The mechanism of action of each drug, if known, can potentially influence the choice of cytotoxicity assays. For example, nitazoxanide inhibits mitochondrial respiration in colorectal cancer spheroids and induces autophagy in macrophages and breast cancer cells [21–23]. Although the mechanism of nitazoxanide activity is not known in HIOs, its potential influence on mitochondrial respiration in these cultures may confound the interpretation for the low viability readings in the metabolism-based assays, but not those that rely on membrane integrity. Oligomycin A is an ATP synthase inhibitor [24], and this activity is in alignment for the low viability with metabolism-based assays. Although auranofin’s mechanism of action is not clearly understood, a known effect is the loss of the mitochondrial membrane potential [25]. How the membrane integrity of HIOs is affected by treatment with auranofin is unclear based on varying results from the two leaky membrane-based cytotoxicity assays. One potential reason for the low metabolism-based assay responses yet higher leaky-membrane assay responses could be that the compounds elicit sublethal cytotoxicity; such non-lethal damage where the cultures remain viable may also add complexity when interpreting cytotoxicity results.

Although these results do not definitively determine which assay is the most accurate for any particular drug, they allow us to generate hypotheses on the differential responses of HIO cultures to these varied compounds and provide a rationale to interpret any accompanying studies, i.e. antiviral activity. It is important to acknowledge certain limitations of this work. We performed our studies primarily as static cultures on 96-well plates, which have different transcriptional profiles compared to the same HIOs plated as transwells, in microfluidic systems, or 3D format [11]. While results were comparable between 2D and 3D HIOs for ritonavir, how generalizable these results are to other drugs, HIO formats or organoids derived from cancer biopsies or induced pluripotent stem cells remains to be determined. Of note, the studies with ritonavir in 3D HIOs were performed with the antiviral added to the wells and the primary basolateral exposure is therefore not entirely representative of how an orally administered drug encounters intestinal epithelial cells. In addition, we only studied HIOs derived from jejunal tissues. In the future, it also will be important to consider how these assays perform in cancer-derived organoids given their increasing use in modeling treatment responses and cell viability for anti-cancer therapeutics [26, 27]. Most of the assays used in this study have been utilized for evaluation of the efficacy of various compounds against patient derived cancer organoids [28]. However, to our knowledge, a comparative study on the performance of these assays in cancer-derived organoids has not been done. Nonetheless, these *in vitro* assays offer a good insight into cell health when doing quick, large-scale screens to discover initial targets. Overall, the variability in results lead us to emphasize the importance of employing multiple assay types for a comprehensive evaluation of cell viability/cytotoxicity for better reliability. Such a recommendation has been made for cancer cell lines previously [29]. Leveraging the strengths of HIOs while being mindful of their limitations enhances the reliability and relevance of cytotoxicity assessments in these cultures.

## Supporting information

S1 File (DOCX)

S1 Data (ZIP)
