# Supplementary material for: Divergent responses of human intestinal organoid monolayers using commercial in vitro cytotoxicity assays
Source: PLoS One. 2024 Jun 10;19(6):e0304526. doi: 10.1371/journal.pone.0304526 (PMC11164375; doi:10.1371/journal.pone.0304526)
Supplement: S1 File — (DOCX) [file pone.0304526.s001.docx]

**
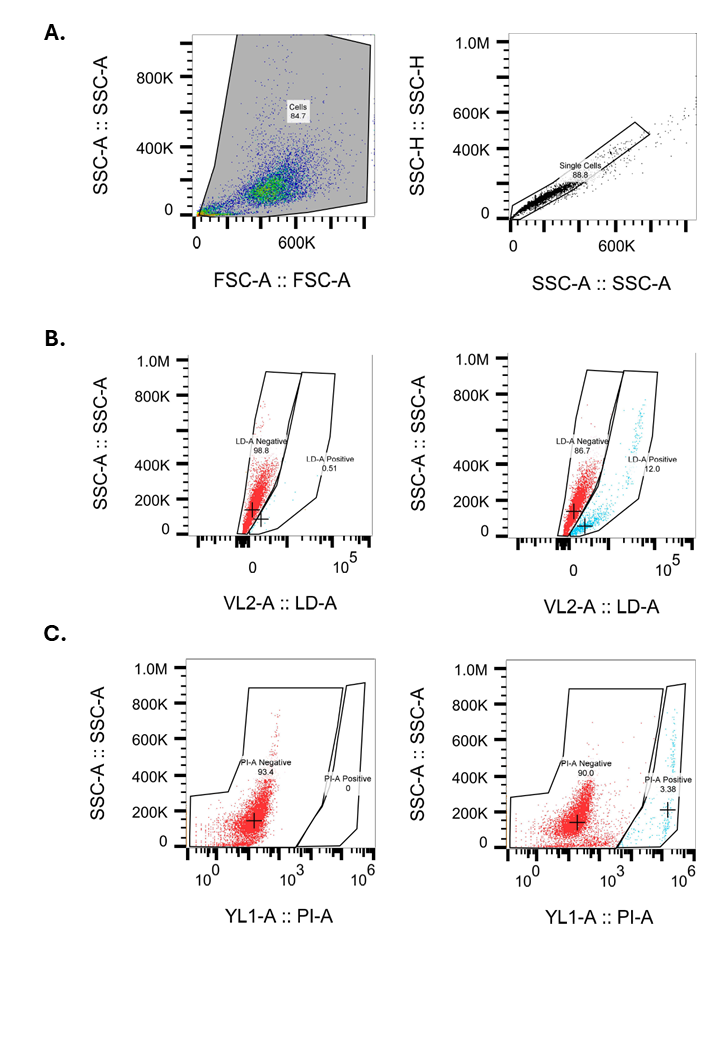
Supporting information for “Divergent responses of human intestinal organoid monolayers using commercial *in vitro* cytotoxicity assays”**

**S1 Fig. Representative gating strategy for evaluation of cytotoxicity by flow cytometry.** Heat-killed cells were used as unstained, single-, and double-stained dye controls. A) The large majority of cells were included and gated for single cells. Gating for the B) LIVE/DEAD (LD) and C) propidium iodide (PI) dyes. Percentages of cells included in the gate are listed on the figures. Final percent viability was based on percent (100 - % cytotoxic)/(total cells captured in gates).


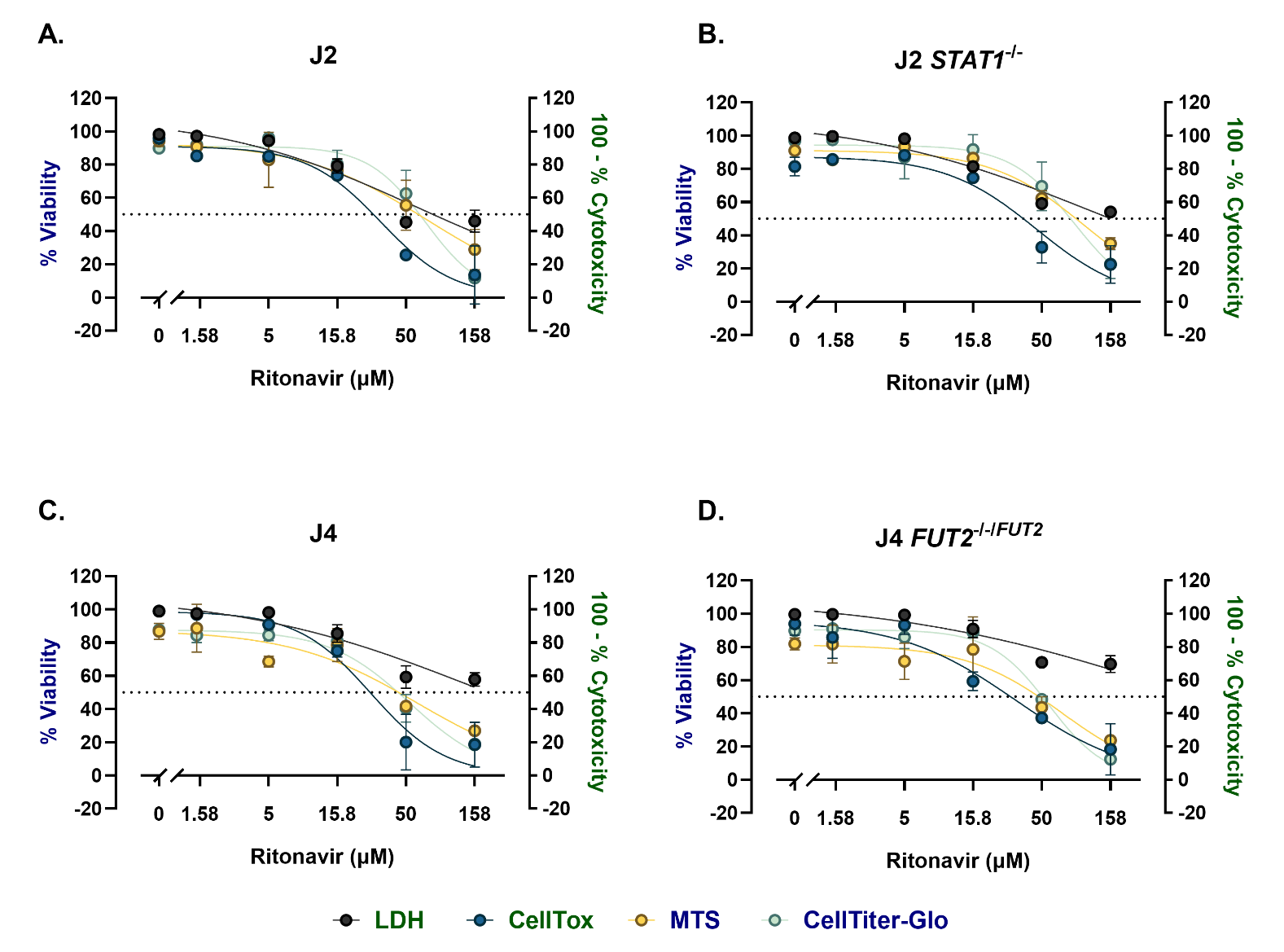


**S2 Fig.** **Variability of plate-based commercial cytotoxicity assays in 3D HIOs.** HIOs were plated in 5 µL Matrigel plugs to confer 3D conformation in 96-well plates. The 3Ds were differentiated for 3 days before treatment. 3D HIOs were treated with vehicle, 1.58, 5, 15.8, 50, or 158 µM ritonavir for 24 h Cytotoxicity was evaluated by the LDH, CellTox, MTS, and CellTiter-Glo assays. Percent viability and 100 minus percent cytotoxicity is shown for the A) J2, B) J2 *STAT1^-/-^* C) J4 D) J4 *FUT2^-/-/FUT2^* HIO lines. 8-10 replicates were used per treatment and data are averaged. The data from LDH and CellTox assasy are plotted on the right y-axis (green) and data from the MTS and CellTiter-Glo assays are plotted on the left y-axis (blue). Data are compiled of n=2 experiments.

**S3 Fig.** **Representative images of ritonavir-treated HIOs.** HIOs were treated with vehicle, 15, 50, and 158 µM ritonavir. Cells were stained with propidium iodide (PI, red) and DAPI (blue). Images are shown for the A) J2, B) J2 *STAT1^-/-^* C) J4 D) J4 *FUT2^-/-/FUT2^* HIO lines. Images are taken at 20X Magnification with an Olympus epifluorescent microscope. Scale bar = 50 µm.
